# The retinal vasculature pathophysiological changes in vision recovery after treatment for indirect traumatic optic neuropathy patients

**DOI:** 10.1007/s00417-021-05208-x

**Published:** 2021-05-11

**Authors:** Yang Gao, Jinmiao Li, Huan Ma, Cong Nie, Xi Lv, Xiaofeng Lin, Guangwei Luo, Jianbo Shi, Rong Lu

**Affiliations:** 1 State Key Laboratory of Ophthalmology, Zhongshan Ophthalmic Center, Sun Yat-Sen University, 54 Xianlie S. Road, Guangzhou, 510060 China; 2 Otorhinolaryngology Hospital, The First Affiliated Hospital of Sun Yat-Sen University, Guangzhou, 510080 China

**Keywords:** Vision recovery, Vasculature, Indirect traumatic optic neuropathy, Retinal oxygen saturation, Optical coherence tomography angiography

## Abstract

**Purpose:**

To evaluate the retinal vasculature pathophysiological changes of indirect traumatic optic neuropathy (ITON) patients after effective surgery.

**Methods:**

Monocular ITON patients who underwent endoscopic trans-ethmosphenoid optic canal decompression (ETOCD) or conservative treatments in Zhongshan Ophthalmic Center from January 2017 to June 2020 were recruited. Visual acuity (VA), visual evoked potential (VEP), oxygen saturation of retinal blood vessels (SO_2_), and optical coherence tomography angiography (OCT-A) were measured. All patients were followed up at least 3 months after treatments.

**Results:**

A total of 95 ITON patients were recruited, including 77 patients who underwent ETOCD and 18 patients who underwent conservative treatments. After treatments, more patients received ETOCD (59/77 = 76.6%) presented with improved VA compared with the patients with conservative treatments (6/18 = 33.3%). Compared with the pre-therapeutic measurements, VEP were significantly improved after surgery in ETOCD-treated patients (*P* < 0.05). Latent periods of P1 and N2, as well as amplitude of P2 of VEP parameters, showed more sensitive to vision recovery (*P* < 0.05). Retinal artery SO_2_ and the differences between arteries and veins were improved in ETOCD-treated patients (*P* < 0.05). Meanwhile, with OCT-A examination, the retinal thickness and retinal vessel density were notably better in ETOCD-treated patients after surgery than that in patients received conservative treatments *(P* < 0.05).

**Conclusions:**

Vision recovery after effective treatment of ITON patients was associated with the increased oxygen saturation of retinal vessels, better availability of oxygen in the retina, greater vessel density, and thicker retinas, which might further underlie the vasculature mechanism of vision recovery in ITON patients. 
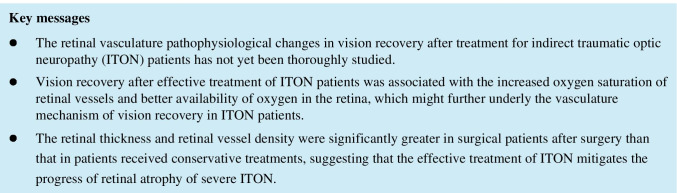

## Introduction

Indirect traumatic optic neuropathy (ITON) is an indirect acute injury of the optic nerve from nonpenetrating effects of craniofacial trauma, which occurs 2–5% of facial trauma and 0.5–2% of head trauma [1–3]. ITON is diagnosed for disruption of visual function with clinical characteristics that include a severe acute decrease of visual acuity, impaired visual evoked potential (VEP), and a relative afferent pupil defect.

The shock wave from concussive forces applied to the head or face is transmitted to the optic canal. It was hypothesized that swelling of the optic nerve within the fixed and limited cavity of the optic canal compromises blood supply, which exacerbates tissue ischemia and causes further damage to the injured optic nerve [4–6]. This pathophysiological hypothesis is commonly cited as a rationale for performing optic canal decompressive surgery. Endoscopic trans-ethmosphenoid optic canal decompression (ETOCD), an effective and less invasive approach of optic canal decompression, has become a trend in recent decades. Various studies reported positive outcomes from ETOCD surgery for ITON cases [7–12]. However, the underlying principle of ETOCD has not yet been proven. The pathophysiological changes after ETOCD, especially the blood supply alteration to the optic nerve and retina, might reveal the underlying mechanism of the effective therapy for ITON.

The mechanism underlying ITON appears to be multifactorial, and vascular insufficiency might be an important factor as in other optic neuropathies. Noninvasive spectrophotometric retinal oximetry has been used to measure oxygen saturation in retinal arteries and veins [13]. Besides, optical coherence tomography angiography (OCT-A) is a noninvasive technique capable of qualitatively and quantitatively evaluating the retinal and choroidal microvascular perfusion. They are both noninvasive diagnostic tools and thereby interesting in evaluating hemodynamic changes in different eye conditions, such as glaucoma, diabetic retinopathy, age-related macular degeneration, and several optic neuropathies [14–23].

In this study, with the retinal oximetry and OCT-A examination, it is feasible to investigate the retinal structural and hemodynamic changes after the effective therapy, thereby revealing the retinal vasculature pathophysiological changes in the process of vision recovery for ITON patients.

## Methods

### Ethics approval

Ethical approval and patient consent were obtained before surgery and the procedures adhered to the tenets of the 1964 Declaration of Helsinki. Protocols were approved by the Institutional Ethics Committee (2019KYPJ155, Medical Ethics Committee, Zhongshan Ophthalmic Center, Guangzhou, Guangdong, China).

### Subjects

This study recruited 95 patients with indirect traumatic optic neuropathy (ITON) in Zhongshan Ophthalmic Center from January 2017 to June 2020, including 77 patients underwent endoscopic trans-ethmosphenoid optic canal decompression (ETOCD) surgery and 18 patients underwent conservative treatments. The inclusion criteria were as follows: (1) patients were diagnosed with ITON; (2) patients’ initial VA ≤ 0.3; (3) patients underwent ETOCD surgery or conservative treatments within 3 months after the injury; (4) patients were followed up for at least 3 months; and (5) patients have no history of other severe ocular disorders or ocular surgery. The written informed consent was acquired from the patients. Details on gender, age, side, lag time from injury to surgery, causes of injuries, blood pressure, intraocular pressure, and axial length were recorded.

The diagnosis of ITON was made by traumatic history and examination, including (1) a closed head injury with no direct trauma to the optic nerve; (2) acute decrease of visual acuity; (3) a positive relative afferent pupil defect (RAPD); and (4) an abnormal visual evoked potential (VEP) with normal fundus examination.

### Treatments

A total 95 patients were divided into the ETOCD group (77 patients) and the conservative group (18 patients) randomly. Doctors have had comprehensively informed the patients about the possible outcomes/risks of treatment options. Treatment for ITON was performed with the patient’s consent.

The indications for ETOCD were as follows: (1) patients were diagnosed with ITON; (2) patients’ initial VA ≤ 0.3; (3) the lag time from the injury to the surgery was less than 3 months; (4) patients have no history of other severe systemic disorders; and (5) the written informed consent was acquired from the patients.

All ETOCD surgeries were performed under general anesthesia. The surgery was performed in the following four steps. (1) Expose the optic canal. (2) Open the optic canal. (3) Incise the annulus of Zinn. (4) Cover the optic nerve with nerve growth factor.

The conservative treatments included observation and neurotrophic drugs.

### Ophthalmic and systemic examination

All subjects underwent comprehensive ocular examinations, including best-corrected visual acuity (BCVA), axial length measurement, non-contact tonometer (NCT), slit-lamp biomicroscopy, fundoscopy, apparent pupillary defect evaluation, and ocular motility assessment. Blood pressure was measured after at least 5 min of resting. Mean arterial blood pressure was computed as diastolic blood pressure plus one-third of pulse pressure.

To evaluate the vision changes after treatments, visual acuity (VA) was measured before and after treatments at various timepoints, i.e., 1 day, 2 days, 3 days, 1 week, 2 weeks, 1 month, and 3 months, respectively. The therapeutic effectiveness was defined by an increase of VA, wider visual fields, and/or brighter visual fields.

### Visual evoked potential (VEP)

Flash VEP was measured before treatments and followed up for 3 months after treatments (RETI-Port, Roland Consult, Brandenburg, Germany).

### Oxygen saturation of retinal blood vessels (SO_2_)

SO_2_ using Oxymap T1 retinal oximeter (Oxymap ehf., Reykjavik, Iceland) was measured before and after treatments in 1 day, 1 week, 2 weeks, 1 month, and 3 months, respectively.

Oxymap T1 is a noninvasive instrument used for measuring in vivo oxygen saturation of retinal arteries (SaO_2_) and veins (SvO_2_), as well as the differences between the arteries and veins (AVD, SaO_2_-SvO_2_). The theoretical basis of Oxymap T1 is the difference in light absorbance of deoxyhemoglobin and oxyhemoglobin at specific wavelengths (570 and 600 nm). SO_2_ values were calculated using a computerized algorithm after collecting fundus images at these two wavelengths. After the participants reached complete cycloplegia, Oxymap examination was conducted following standard procedures (version from November 21, 2013).

### Optical coherence tomography angiography (OCT-A)

All subjects were examined under a single OCT-A system (AngioVue; Optovue, Inc., Fremont, CA, USA), which was able to visualize and quantify the microvasculature in the retina. The scanning speed of the device was 70,000 A-scans per second, and the central wavelength was 840 nm. Each studied eye underwent a 3.0 × 3.0 mm cube angio scan centered on the optic nerve head and a 3.0 × 3.0 mm macular cube angio scan centered on the fovea. A split spectrum amplitude decorrelation angiography algorithm was applied to produce OCT-A images.

Vessel density was defined as the percentage of perfused vascular area in relation to the whole selected region in en face views. All vessel density parameters were automatically calculated using the built-in program. Peripapillary vessel density parameters were obtained from the 3.0 × 3.0 mm cube angio scan centered on the optic nerve head. The macular whole image vessel density was generated from the 3.0 × 3.0 mm cube angio scan centered on the fovea. The optic nerve head scanning area was further divided into the inside disc and peripapillary fields. The macular scanning area was segmented into three areas: fovea, parafoveal, and perifoveal zone. The parafoveal and perifoveal zone was further divided into 4 sessions by an annular grid: temporal, superior, nasal, and inferior.

Apart from vessel density indices, thickness of the retinal nerve fiber layer (RNFL) of optic nerve head area was measured automatedly. The optic nerve head scanning area was divided into 4 sessions by an annular grid: temporal, superior, nasal, and inferior.

### Statistical analysis

Examination operators for VEP, SO_2_, and OCT-A as well as the statistician were blinded from patients’ details and treatment groups.

Continuous variables were described as means ± standard deviation (SD). A paired *t* test was performed to compare patients’ VA, VEP measures, retinal oxygen saturation, and OCT-A measurements before and after treatments. A *t* test was performed to compare the parameters between different groups, and a Bonferroni correction was applied if necessary. Statistical analysis was performed with statistical software (SPSS version 20; IBM Corp., Armonk, NY, USA). Statistical significance was defined with *P* value of less than 0.05.

## Results

### Baseline characteristics of patients

A total of 95 patients with monocular indirect traumatic optic neuropathy (ITON), including 77 patients treated with endoscopic trans-ethmosphenoid optic canal decompression (ETOCD) surgery and 18 patients who underwent conservative treatments, were recruited in the study. Participant characteristics are listed in Table 1. In 77 ETOCD-treated patients, 68 were male (88.3%) and 9 were female (11.7%). The mean age of all patients was 26.9 ± 14.1 years old (range 5–62). The affected eyes were 38 right eyes and 39 left eyes. To avoid the patients’ selection bias, the initial VA, lag time from the injury to the therapy, sex, age, and causes of injuries of these two treatment groups were analyzed statistically using *t* test. And these parameters between these two groups were matched (all *P* > 0.05; Table 1).

**Table 1:** Clinical characteristics of 95 enrolled subjects and included eyes

Variables	ETOCD	Conservative treatment
Patients	77	18
Sex, male	68 (88.3%)	16 (88.9%)
Age (years)	26.9 ± 14.1 (5–62)	29.0 ± 17.8 (10–59)
Side, right	38 (49.4%)	9 (50.0%)
Lag time (days)	20.0 ± 16.9 (3–80)	23.2 ± 16.0 (3–62)
Causes of injuries		
Traffic accidents	46 (59.7%)	10 (55.6%)
Falls	21 (27.3%)	6 (33.3%)
Fights/assaults	10 (13.0%)	2 (11.1%)
Initial VA		
VA, range	NLP to 0.3	NLP to 0.3
logMAR VA	− 3.38 ± 1.42	− 3.45 ± 1.67
SBP (mmHg)	121.7 ± 9.2 (92–156)	122.6 ± 10.7 (94–156)
DBP (mmHg)	75.5 ± 7.2 (56–95)	75.1 ± 6.3 (56–92)
MABP (mmHg)	90.9 ± 7.3 (74–125)	90.6 ± 7.1 (74–123)
IOP (mmHg)	14.6 ± 3.7 (7.7–32)	14.3 ± 5.1 (8–32)
Axial length (mm)	23.6 ± 0.7 (21.3–26.4)	23.5 ± 0.7 (21.3–26.4)

*ETOCD*, endoscopic trans-ethmosphenoid optic canal decompression; lag time, lag time from the injury to the treatment; *VA*, visual acuity; *SBP*, systolic blood pressure; *DBP*, diastolic blood pressure; *MABP*, mean arterial blood pressure; *IOP*, intraocular pressure

### Vision was efficiently improved in ITON patients after surgery

After ETOCD, 76.6% (59/77) patients achieved VA improvement. Meanwhile, the percentage of patients with VA improvement after the conservative treatment was 33.3% (6/18). The effectiveness was defined by increased VA, wider visual fields, or even brighter visual fields.

The proportions of VA improvement in total 77 ITON patients were timely increasing at 58.4, 66.2, 67.5, 70.1, 75.3, and 76.6% in 1 day (D1), 2 days (D2), 3 days (D3), 1 week (W1), 1 month (M1), and 3 months (M3), respectively. This clinical observation shows the rapid and steady effectiveness of ETOCD in ITON patients.

There was no difference between the initial logMAR VA before treatments of two groups (ETOCD group, − 3.45 ± 1.42; conservative treatment group, − 3.38 ± 1.67; *P* > 0.05). Nevertheless, after 3 months of two different therapies, logMAR VA was significant between ETOCD group and conservative treatment group (− 2.48 ± 1.61 vs − 3.13 ± 1.72, *P* < 0.05).

### The visual evoked potential (VEP) was significantly improved in ITON patients treated with ETOCD surgery, especially in patients with vision recovery after surgery

The latent periods of N1, N2, and P1 for VEP were significantly decreased, and the amplitudes of P1 and P2 were significantly increased after ETOCD surgery in all ITON patients (all *P* < 0.001, Table 2), and these above parameters after treatments of ETOCD-treated patients were significantly better than those of conservative therapeutic patients, suggesting VEP was significantly improved after ETOCD.

**Table 2:** The flash visual evoked potential (VEP) of indirect traumatic optic neuropathy (ITON) patients before and 3 months after endoscopic trans-ethmosphenoid optic canal decompression (ETOCD) and the conservative treatment

Variables	Groups	Time points		
		Pre-treatment	Post-treatment	*P*
Latent period of N1 (ms)	ETOCD-Improved VA	69.60 ± 14.44	50.21 ± 20.69	< 0.001*
	ETOCD-No improved VA	76.00 ± 19.44	54.01 ± 18.51	< 0.001*
	*P*^*†*^	0.085	0.308	
	ETOCD-Total	71.13 ± 18.92	51.12 ± 16.74	< 0.001*
	Conservative treatment	69.70 ± 19.17	66.24 ± 17.58	0.211
	*P*^*#*^	0.756	0.001*	
Latent period of N2 (ms)	ETOCD-Improved VA	111.57 ± 20.37	82.01 ± 18.35	< 0.001*
	ETOCD-No improved VA	114.51 ± 11.25	107.43 ± 10.62	0.428
	*P*^*†*^	0.245	< 0.001*	
	ETOCD-Total	111.80 ± 19.69	87.51 ± 14.13	< 0.001*
	Conservative treatment	109.25 ± 15.98	100.48 ± 24.59	0.050
	*P*^*#*^	0.714	< 0.001*	
Latent period of P1 (ms)	ETOCD-Improved VA	89.01 ± 16.90	65.63 ± 18.74	< 0.001*
	ETOCD-No improved VA	92.11 ± 11.09	90.94 ± 22.43	0.347
	*P*^*†*^	0.301	0.009*	
	ETOCD-Total	89.50 ± 16.77	72.30 ± 27.53	< 0.001*
	Conservative treatment	88.47 ± 15.36	85.57 ± 25.36	0.397
	*P*^*#*^	0.847	0.013*	
Latent period of P2 (ms)	ETOCD-Improved VA	128.31 ± 25.51	128.42 ± 26.02	0.987
	ETOCD-No improved VA	129.77 ± 15.19	130.93 ± 23.98	0.765
	*P*^*†*^	0.715	0.662	
	ETOCD-Total	128.65 ± 20.81	129.02 ± 24.82	0.207
	Conservative treatment	125.69 ± 22.58	123.89 ± 26.71	0.514
	*P*^*#*^	0.152	0.101	
Amplitude of P1 (μV)	ETOCD-Improved VA	3.42 ± 1.41	6.38 ± 3.58	< 0.001*
	ETOCD-No improved VA	4.48 ± 2.31	7.20 ± 4.09	0.001*
	*P*^*†*^	0.137	0.108	
	ETOCD-Total	3.67 ± 2.71	6.58 ± 4.16	< 0.001*
	Conservative treatment	4.01 ± 2.16	4.28 ± 3.12	0.769
	*P*^*#*^	0.514	< 0.001*	
Amplitude of P2 (μV)	ETOCD-Improved VA	4.60 ± 1.91	6.92 ± 3.02	< 0.001*
	ETOCD-No improved VA	4.92 ± 1.37	4.43 ± 2.83	0.291
	*P*^*†*^	0.196	0.006*	
	ETOCD-Total	4.69 ± 1.46	6.07 ± 3.21	< 0.001*
	Conservative treatment	4.93 ± 2.47	4.81 ± 4.19	0.844
	*P*^*#*^	0.845	0.041*	

Data are the means plus/minus standard deviations. *ETOCD*, endoscopic trans-ethmosphenoid optic canal decompression; *VA*, visual acuity; *P, P* value between values of pre-treatment and post-treatment; *P*^*†*^*, P* value between improved VA group and no improved VA group after ETOCD; *P*^*#*^*, P* value between two groups treated with ETOCD and the conservation treatment; *P < 0.05

Moreover, to explore if VEP change was related to surgical effectiveness, based on surgery effectiveness, ITON patients were divided into two groups: with (*n* = 59) or without (*n* = 18) VA improvement after ETOCD (Table 2). All VEP parameters, including the latent period of N1, N2, P1, and P2, as well as the amplitude of P1 and P2, were not statistically different between these two groups before ETOCD. Notably, in patients with VA improvement, the latent period of P1, the latent period of N2, and the amplitude of P2 were improved significantly (decreased from 89.01 ± 16.90 to 65.63 ± 18.74 ms, decreased from 111.57 ± 20.37 to 82.01 ± 18.35 ms, and increased from 4.60 ± 1.91 to 6.92 ± 3.02 μV, respectively, all *P* < 0.001), and they were not significantly improved in patients without VA improvement. The results discovered that above three VEP parameters were critical and significantly related to VA improvement after ETOCD. Nevertheless, VEP results were not of correlation with age, gender, side of injury, and courses of injuries (*P* > 0.05).

### The retinal oxygen saturation was significantly improved in ITON patients treated with ETOCD surgery, especially in patients with vision recovery after surgery

**Fig. 1:**
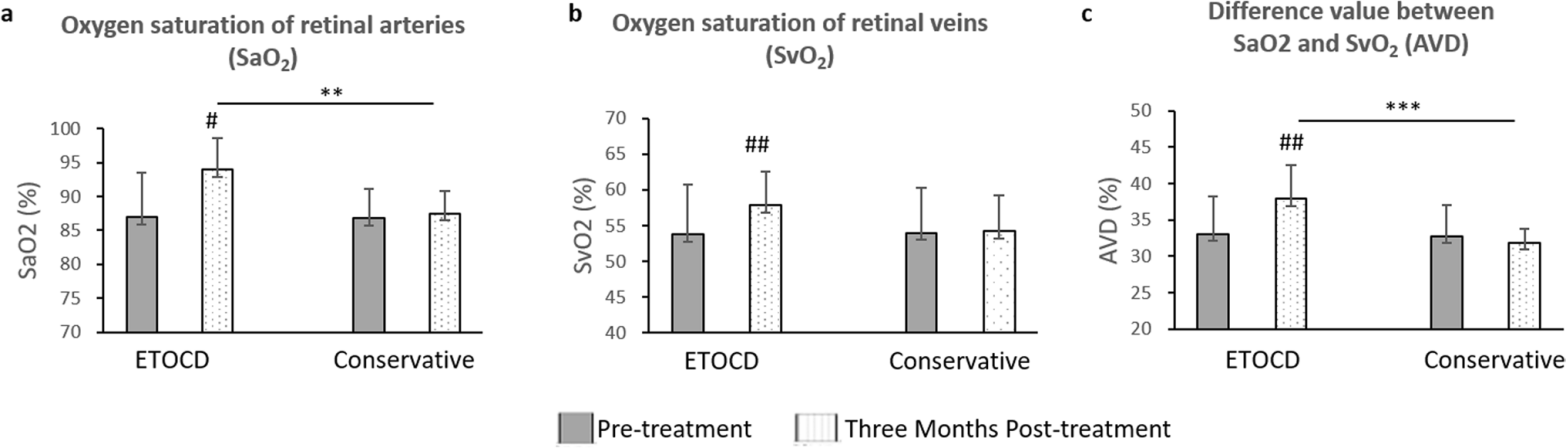
Oxygen saturation of retinal blood vessels of indirect traumatic optic neuropathy (ITON) patients before and 3 months after endoscopic trans-ethmosphenoid optic canal decompression (ETOCD) and the conservative treatment. **a** SaO_2_, **b** SvO_2_, and **c** AVD before and after treatments in ETOCD-treated patients and conservative therapeutic patients, respectively. ^#^*P* < 0.05; ^##^*P* < 0.01 between values of pre-treatment and 3 months post-treatment. **P < 0.01; ***P < 0.001 between values of post-ETOCD and post-conservative therapy. SaO_2_, oxygen saturation of retinal arteries; SvO_2_, oxygen saturation of retinal veins; AVD, the difference value between SaO_2_ and SvO_2_

The oxygen saturation of retinal arteries (SaO_2_) and veins (SvO_2_) and the differences between the arteries and veins (AVD, SaO_2_-SvO_2_) were measured as the main outcomes. For the patients underwent ETOCD surgery, compared to the pre-operative SaO_2_ (86.91 ± 6.63%), the SaO_2_ increased significantly throughout D1 (88.97 ± 6.25%, *P* = 0.002) to M3 (93.94 ± 4.69%, *P* < 0.001) in all ITON patients after ETOCD surgery (Fig. 1a). Similarly, the post-operative SvO_2_ increased significantly throughout W1 (57.17 ± 5.36%, *P* < 0.001) to M3 (57.82 ± 4.67%, *P* < 0.001), compared with pre-operative SvO_2_ (53.79 ± 7.00%) (Fig. 1b). The pre-operative AVD was 33.07 ± 5.08%, and the post-operative AVD increased significantly throughout D1 (33.91 ± 7.16%, *P* = 0.004) to M3 (37.91 ± 4.57%, *P* < 0.001) (Fig. 1c).

However, for the patients treated with conservative therapy, there was no difference between these above parameters of pre-treatment and post-treatment (Fig. 1). Besides, SaO_2_ (Fig. 1a) and AVD (Fig. 1c) of ETOCD-treated patients 3 months after the surgery were significantly improved than that of conservative therapeutic patients (*P* = 0.003 and *P* < 0.001, respectively).

Meanwhile, the diameter and length of arteries and veins did not show any difference between pre-therapeutic and post-therapeutic measurements in both treatment groups (*P* > 0.05, Table 3).

**Table 3:** Diameter and length of retinal blood vessels in indirect traumatic optic neuropathy (ITON) patients before and 3 months after endoscopic trans-ethmosphenoid optic canal decompression (ETOCD) and the conservative treatment

Variables	Groups	Time points		
		Pre-treatment	Post-treatment	*P*
Diameter of arteries	ETOCD-Improved VA	11.86 ± 1.29	11.01 ± 1.45	0.054
	ETOCD-No improved VA	12.44 ± 2.68	10.75 ± 1.25	0.112
	*P*^*†*^	0.245	0.293	
	ETOCD-Total	12.00 ± 1.45	10.95 ± 2.40	0.100
	Conservative treatment	12.04 ± 2.14	10.89 ± 2.59	0.100
	*P*^*#*^	0.847	0.798	
Diameter of veins	ETOCD-Improved VA	16.12 ± 2.31	15.54 ± 2.05	0.072
	ETOCD-No improved VA	15.98 ± 2.31	13.36 ± 1.78	0.212
	*P*^*†*^	0.729	0.086	
	ETOCD-Total	16.09 ± 2.30	15.02 ± 2.70	0.112
	Conservative treatment	16.89 ± 2.59	15.48 ± 1.79	0.278
	*P*^*#*^	0.347	0.654	
Length of arteries	ETOCD-Improved VA	2373.89 ± 1153.14	2705.67 ± 658.829	0.031
	ETOCD-No improved VA	2599.75 ± 1422.22	2262.50 ± 859.50	0.050
	*P*^*†*^	0.591	0.472	
	ETOCD-Total	2428.10 ± 1248.29	2599.31 ± 801.26	0.102
	Conservative treatment	2523.14 ± 1312.25	2565.66 ± 924.02	0.854
	*P*^*#*^	0.512	0.821	
Length of veins	ETOCD-Improved VA	2562.22 ± 730.41	2810.33 ± 733.80	0.334
	ETOCD-No improved VA	2398.00 ± 736.23	2607.00 ± 556.34	0.219
	*P*^*†*^	0.051	0.776	
	ETOCD-Total	2522.807 ± 756.54	2761.53 ± 568.80	0.147
	Conservative treatment	2489.26 ± 893.36	2619.26 ± 783.25	0.358
	*P*^*#*^	0.379	0.569	

Data are the means plus/minus standard deviations. *ETOCD*, endoscopic trans-ethmosphenoid optic canal decompression; *VA*, visual acuity; *P*, *P* value between values of pre-treatment and post-treatment; *P*^†^, *P* value between improved VA group and no improved VA group after ETOCD; *P*^#^, *P* value between two groups treated with ETOCD and the conservation treatment

**Fig. 2:**
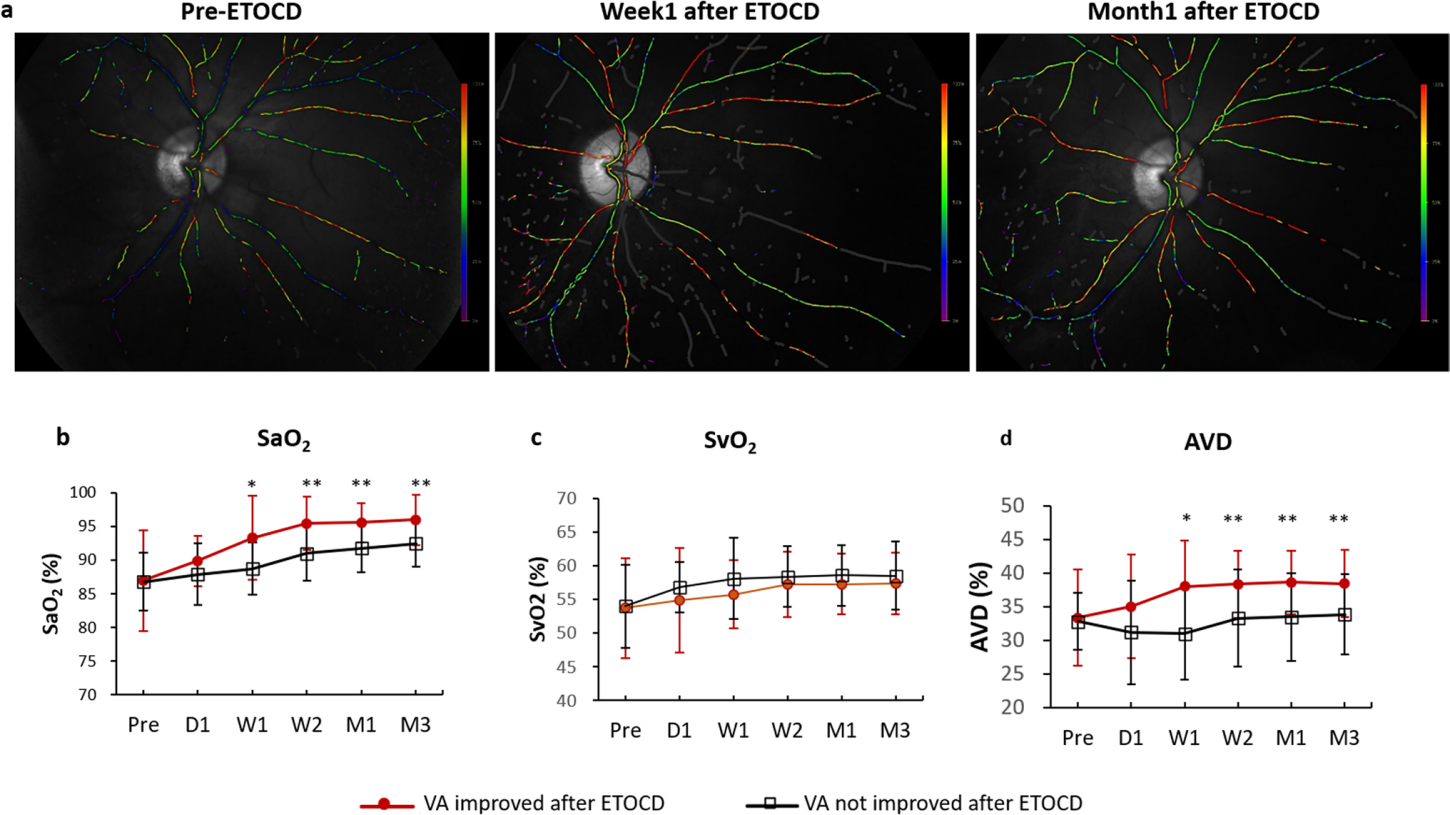
The oxygen saturation of retinal vessels of indirect traumatic optic neuropathy (ITON) patients was improved after endoscopic trans-ethmosphenoid optic canal decompression (ETOCD). **a** The typical retinal oxygen saturation images of ITON patients before ETOCD (left), 1 week (middle) and 1 month after ETOCD (right). The redder the color, the higher the oxygen saturation of retinal vessels. Throughout follow-up timepoints from D1 to M3, **b** SaO_2_ was significantly higher in patients with VA improvement than in those without VA improvements, *P < 0.05; **P < 0.01; **c** SvO_2_ was not significantly different before and after ETOCD; and **d** AVD was significantly higher in patients with VA improvement than in those without VA improvements, *P < 0.05; **P < 0.01. Post-surgery timepoints: after 1 day (D1), 1 week (W1), 2 weeks (W2), 1 month (M1), and 3 months (M3)

Moreover, to explore if the change of oxygen saturation of retinal vessels was related to VA improvement, we divided all ITON patients into two groups based on vision recovery after surgery (Fig. 2). While compared between the two groups, SaO_2_ was found significantly increased with VA-improved group, from W1 (93.30 ± 6.24 vs 88.13 ± 3.90, *P* = 0.03) to M3 (95.04 ± 2.94 vs 91.76 ± 3.62, *P* < 0.01, Fig. 2b,). Notably, AVD, which may imply the use of oxygen by cells, was also significantly increased in patients with VA improvement than those without, throughout W1 (38.80 ± 6.90 vs 31.53 ± 6.88, *P* = 0.02) to 3 months (38.45 ± 4.96 vs 27.87 ± 1.96, *P* < 0.01, Fig. 2d). However, for other parameters after ETOCD, including SvO_2_ as well as the diameter and length of arteries and veins, there was no significant difference between patients with or without post-operative VA improvement (Fig. 2c).

Meanwhile, the diameter and length of arteries and veins did not show any difference between ETOCD-treated patients with and without VA improvement after the surgery (*P* > 0.05, Table 3). These measures of the retinal oxygen saturation did not show any correlation with age, gender, side of injury, and causes of injuries (*P* > 0.05).

### VA improvement is associated with thicker retinas and greater vessel density in ITON patients after the surgery

**Fig. 3:**
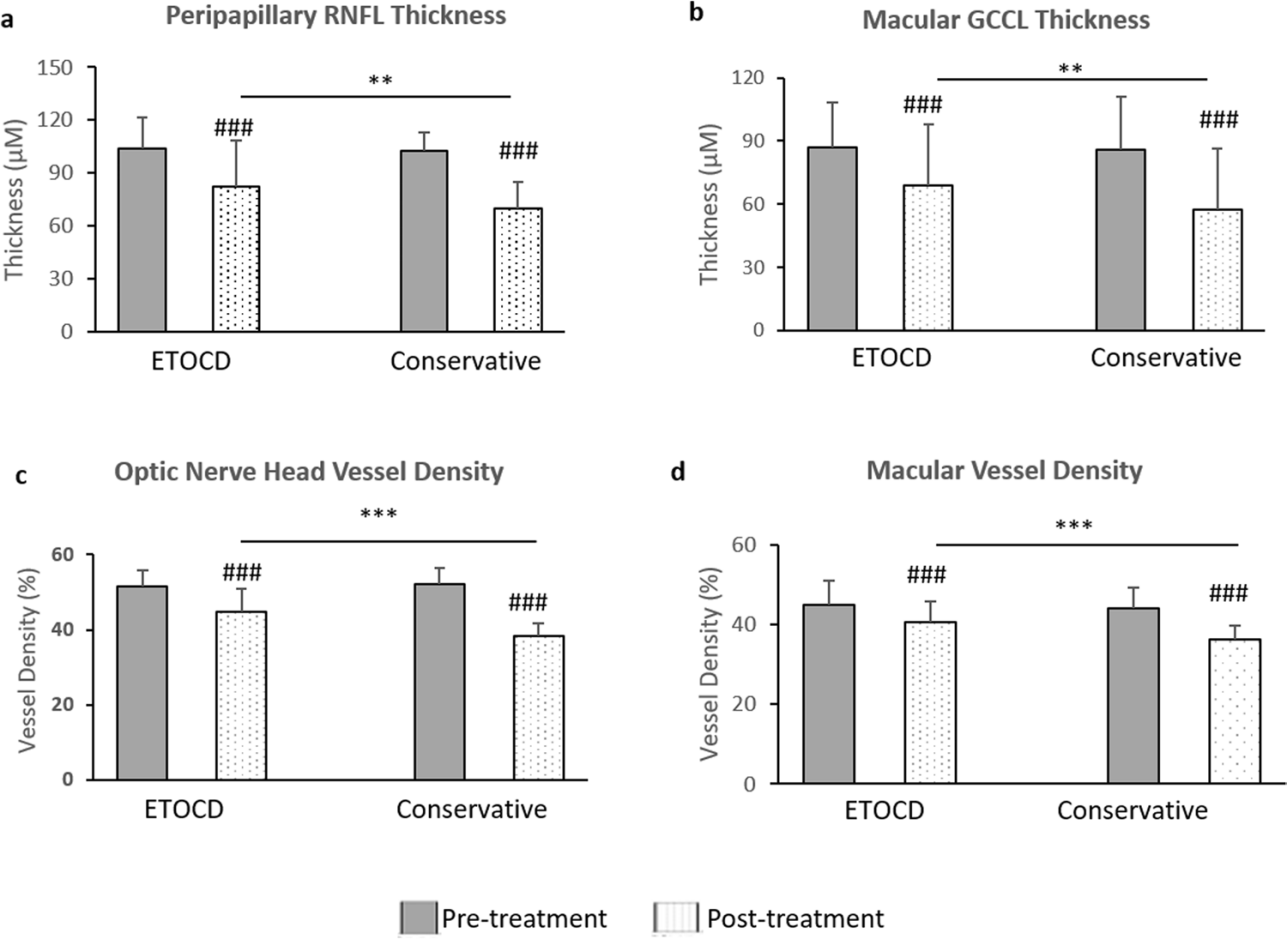
The thickness and vessel density in optic nerve head area and macular between indirect traumatic optic neuropathy (ITON) patients before and 3 months after endoscopic trans-ethmosphenoid optic canal decompression (ETOCD) and the conservative treatment. Comparison of **a** the thickness of peripapillary retinal nerve fiber layer (RNFL), **b** the thickness of macular ganglion cell complex (GCC), **c** the retinal vessel density of optic nerve head area, and** d** the retinal vessel density of macular before (gray) and after (dots) treatments, and between groups treated with ETOCD (left) and the conservative treatment (right). ^###^*P* < 0.001 between values of pre-treatment and 3 months post-treatment. **P < 0.01; ***P < 0.001 between values of post-ETOCD and post-conservative therapy

**Fig. 4:**
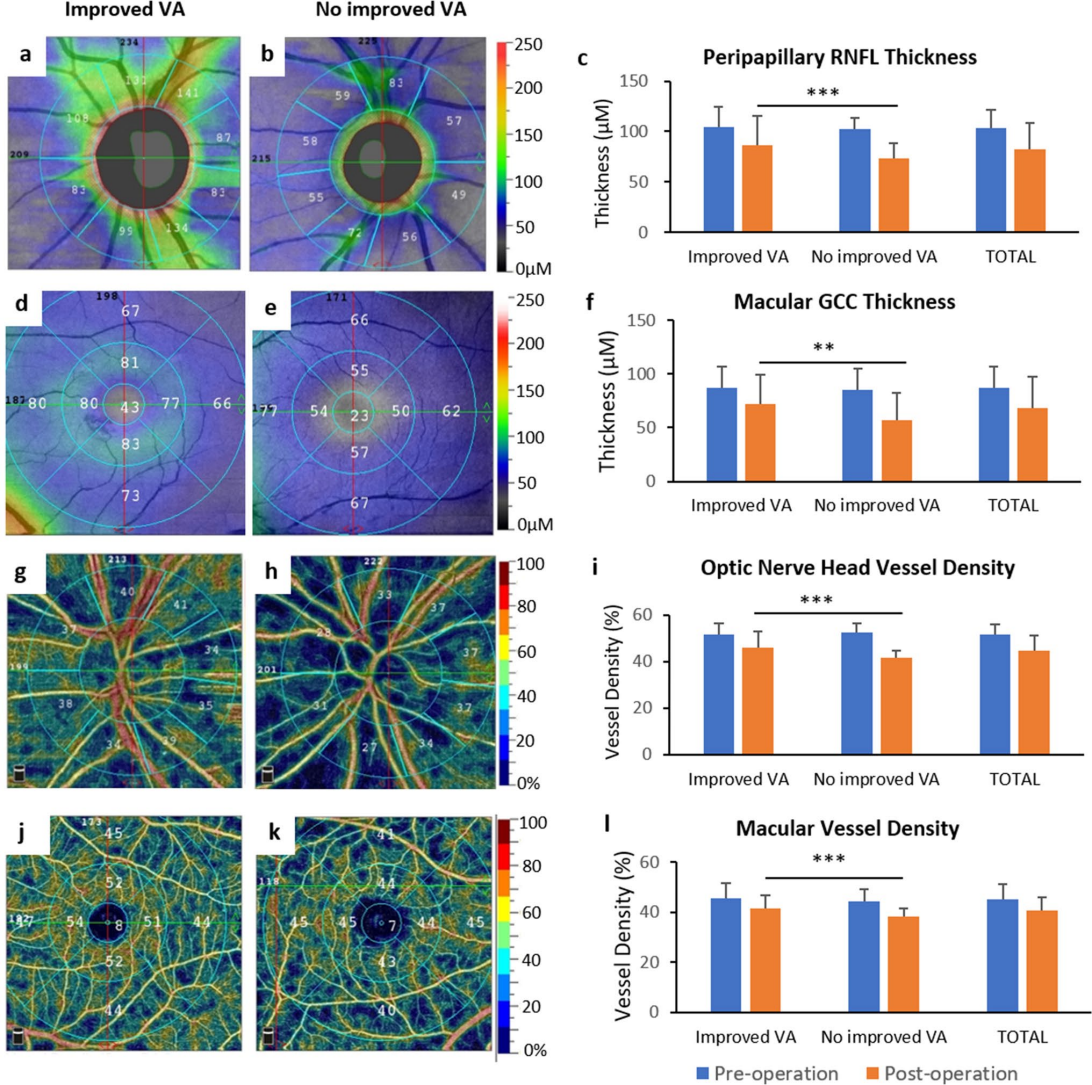
The retinal thickness and vessel density in optic nerve head area and macular between indirect traumatic optic neuropathy (ITON) patients with and without VA improvements after endoscopic trans-ethmosphenoid optic canal decompression (ETOCD). **(a–b)** The typical image of the thickness of peripapillary retinal nerve fiber layer (RNFL) in patients with **(a)** and without **(b)** post-operative improved VA after ETOCD. **(c)** Comparison of peripapillary RNFL before (blue) and after (orange) ETOCD surgery, and between groups of patients with (left) and without (middle) VA improvements. **(d–e)** The typical image of the thickness of macular ganglion cell complex (GCC) in patients with (d) and without **(e)** post-operative improved VA after ETOCD. **(f)** Comparison of macular (GCC) before (blue) and after (orange) ETOCD surgery, and between groups of patients with (left) and without (middle) VA improvements. **(g–h)** The typical image of retinal vessel density in optic nerve head area of patients with **(g)** and without **(h)** post-operative improved VA after ETOCD. **(i)** Comparison of retinal vessel density in optic nerve head area before (blue) and after (orange) ETOCD surgery, and between groups of patients with (left) and without (middle) VA improvements. **(j–k)** The typical image of retinal vessel density in macular of patients with **(j)** and without **(k)** post-operative improved VA after ETOCD. **(l)** Comparison of retinal vessel density in macular before (blue) and after (orange) ETOCD surgery, and between groups of patients with (left) and without (middle) VA improvements. **P < 0.01; ***P < 0.001 between the post-operative parameter for two groups of patients with and without VA improvements

The retina thickness and the vessel density of optic nerve head area and macular before and 3 months after treatments were measured as the main outcomes using the optical coherence tomography angiography (OCT-A; Table 4, Figs. 3 and 4).

**Table 4:** Retinal thickness and vessel density of optic nerve head and macular in indirect traumatic optic neuropathy (ITON) patients before and 3 months after endoscopic trans-ethmosphenoid optic canal decompression (ETOCD)

Variables	ETOCD			Conservative treatment				*P*-pre		*P*-post	
	Pre-treatment	Post-treatment	*P*	Pre-treatment		Post-treatment	*P*				
Retinal nerve fiber layer											
Peripapillary	103.70 ± 17.93	82.07 ± 26.51	< 0.001*	102.69 ± 10.43		69.93 ± 14.63	< 0.001*	0.642		0.005*	
Temporal	73.15 ± 16.15	65.76 ± 18.38	< 0.001*	72.50 ± 7.64		60.20 ± 4.40	< 0.001*	0.341		0.021*	
Superior	128.27 ± 28.20	95.79 ± 43.72	< 0.001*	129.55 ± 14.86		90.73 ± 26.97	< 0.001*	0.701		< 0.001*	
Nasal	89.44 ± 16.90	73.30 ± 18.26	< 0.001*	92.17 ± 14.66		64.46 ± 7.26	< 0.001*	0.257		0.004*	
Inferior	127.64 ± 23.65	94.74 ± 36.46	< 0.001*	133.33 ± 18.49		89.41 ± 25.01	< 0.001*	0.177		< 0.001*	
Ganglion cell complex layer											
Whole image	86.82 ± 21.35	68.69 ± 29.35	< 0.001*	85.82 ± 25.35		57.22 ± 29.26	< 0.001*		0.913		0.009*
Fovea	46.59 ± 17.91	38.55 ± 19.84	< 0.001*	47.12 ± 18.59		33.03 ± 20.35	< 0.001*		0.721		0.031*
ParaFovea	92.46 ± 25.68	78.33 ± 30.25	< 0.001*	92.97 ± 27.89		68.87 ± 31.28	< 0.001*		0.851		0.002*
PeriFovea	91.91 ± 19.53	74.53 ± 28.39	< 0.001*	91.73 ± 21.47		60.31 ± 29.78	< 0.001*		0.918		0.001*
Optic nerve head vessel density											
ONH-wiVD	51.66 ± 4.31	44.69 ± 6.40	< 0.001*	52.22 ± 4.10	38.47 ± 3.11		< 0.001*		0.325		< 0.001*
Cap ONH-wiVD	45.91 ± 4.89	37.68 ± 7.07	< 0.001*	46.50 ± 2.76	31.30 ± 3.04		< 0.001*		0.379		< 0.001*
ID ONH-wiVD	53.18 ± 3.14	50.12 ± 4.33	< 0.001*	53.97 ± 4.12	45.55 ± 3.15		< 0.001*		0.217		< 0.001*
ID Cap ONH-wiVD	42.73 ± 3.12	39.20 ± 5.23	< 0.001*	43.65 ± 2.06	33.81 ± 4.98		< 0.001*		0.562		< 0.001*
Peri ONH-wiVD	53.18 ± 5.63	44.04 ± 7.48	< 0.001*	52.61 ± 1.67	37.74 ± 3.70		< 0.001*		0.487		< 0.001*
Peri Cap ONH-wiVD	47.34 ± 6.80	36.96 ± 8.33	< 0.001*	46.65 ± 7.53	29.35 ± 3.52		< 0.001*		0.278		< 0.001*
Macular vessel density											
m-wiVD	45.05 ± 6.00	40.65 ± 5.13	< 0.001*	44.17 ± 5.02	36.29 ± 3.39		< 0.001*		0.161		< 0.001*
Fovea	16.50 ± 9.02	12.86 ± 5.71	< 0.001*	15.37 ± 5.90	8.14 ± 3.94		< 0.001*		0.088		< 0.001*
Parafovea	46.45 ± 6.34	43.96 ± 4.45	0.001*	46.43 ± 5.82	39.82 ± 3.87		< 0.001*		0.798		0.021*
Temporal	45.76 ± 7.58	43.89 ± 4.93	0.022*	45.09 ± 5.82	38.33 ± 3.87		< 0.001*		0.818		0.002*
Superior	47.17 ± 6.18	44.60 ± 4.11	0.001*	46.69 ± 5.82	41.33 ± 3.87		< 0.001*		0.644		0.001*
Nasal	46.49 ± 7.16	44.71 ± 5.57	0.029*	46.39 ± 7.29	39.35 ± 3.23		< 0.001*		0.178		0.034*
Inferior	46.39 ± 7.61	42.65 ± 5.93	< 0.001*	47.55 ± 6.54	40.25 ± 2.89		< 0.001*		0.506		0.657
Perifovea	46.00 ± 6.38	42.70 ± 5.37	< 0.001*	45.39 ± 5.84	37.09 ± 2.91		< 0.001*		0.071		0.005*
Temporal	43.65 ± 5.65	39.25 ± 5.03	< 0.001*	44.51 ± 5.39	36.61 ± 3.28		< 0.001*		0.891		0.030*
Superior	46.39 ± 6.73	40.40 ± 5.58	< 0.001*	45.89 ± 4.64	35.63 ± 3.59		< 0.001*		0.142		< 0.001*
Nasal	47.05 ± 7.71	42.72 ± 6.57	< 0.001*	47.78 ± 5.91	38.65 ± 4.89		< 0.001*		0.281		0.039*
Inferior	46.20 ± 7.88	40.35 ± 5.70	< 0.001*	45.14. ± 5.04	37.45 ± 2.99		< 0.001*		0.214		0.097
FAZ area	0.32 ± 0.17	0.29 ± 0.10	0.269	0.32 ± 0.04	0.32 ± 0.12		0.753		0.995		0.228

Data are the means plus/minus standard deviations. *ONH*, optic nerve head; *Wi*, whole image; *VD*, vessel density; *Cap*, capillary; *ID*, inside disc; *FAZ*, foveal avascular zone. *P*, *P* value between measurements of pre-operation and post-operation with the same therapy; *P*-pre, *P* value between measurements of two groups with different therapies before treatments; *P*-post, *P* value between measurements of two groups with different therapies after treatments

For the patients underwent ETOCD surgery, compared to the pre-operative peripapillary thickness of peripapillary retinal nerve fiber layer (RNFL) (103.70 ± 17.93), the post-operative thickness of RNFL significantly decreased 3 months after ETOCD (82.07 ± 26.51; *P* < 0.001; Fig. 3a, Table 4) in ITON patients. Meanwhile, the post-operative macular ganglion cell complex (GCC; 68.69 ± 29.35) was significantly thinner than the pre-operative GCC (86.82 ± 21.35; *P* < 0.001; Fig. 3b, Table 4). Besides, the post-operative vessel density parameters of optic nerve head area decreased significantly 3 months after ETOCD compared with pre-operation, including whole image, inside disk, and radial peripapillary capillary vessel density (all *P* < 0.001; Fig. 3c, Table 4). Similar to the decreasing tendencies of the vessel density in optic nerve head area, the difference of macular vessel density whole image (m-wiVD) and in each grid session was statistically significant (*P* < 0.05; Fig. 3d, Table 4), expect foveal avascular zone (FAZ, *P* = 0.269; Table 4).

Meanwhile, for the patients treated with conservative therapy, above OCT-A parameters were decreased significantly 3 months after the treatment compared with pre-treatment (all *P* < 0.001; Fig. 3, Table 4), expect FAZ (*P* = 0.753; Table 4). Notably, while compared between the two treatment groups after two different therapies, most parameters were found significantly greater in ETOCD-treated group ((peripapillary RNFL thickness: 82.07 ± 26.51 vs 69.93 ± 14.63; macular GCC thickness: 68.69 ± 29.35 vs 57.22 ± 29.26; radial peripapillary capillary whole image vessel density: 44.69 ± 6.40 vs 38.47 ± 3.11; macular whole image vessel density: 40.65 ± 5.13 vs 36.29 ± 3.39; all *P* < 0.01; Fig. 3a–d).

Again, to explore whether the change of vessel density was related to surgical effectiveness, the ITON patients were divided into two groups based on improved VA or not after surgery. Before surgery, the retina thickness and the vessel density of optic nerve head area and macular were not significantly different between the two groups (all *P* > 0.05). Notably, while compared between the two groups after surgery, most parameters were found significantly greater in VA-improved group (peripapillary RNFL thickness: 85.99 ± 29.14 vs 72.93 ± 15.96 [Fig. 4a–c]; macular GCC thickness: 72.26 ± 27.59 vs 57.31 ± 25.47 [Fig. 4d–f]; radial peripapillary capillary whole image vessel density: 46.06 ± 6.95 vs 41.48 ± 3.04 [Fig. 4g–i]; macular whole image vessel density: 41.34 ± 5.31 vs 38.27 ± 3.15 [Fig. 4j–l]; all *P* < 0.01). These results showed that VA improvement after surgery related with both retinal thickness and vessel density, which indicated effective surgery, may mitigate the retinal atrophy after severe ITON.

## Discussion

In the present study, we investigated the retinal vasculature alteration in indirect traumatic optic neuropathy (ITON) patients, and found vision recovery after the effective therapy for ITON was associated with the increased oxygen saturation of retinal vessels and the better availability of oxygen in the retina. Moreover, ITON patients who got vision recovery after the effective therapy also presented with thicker retinas and better vessel density, which indicated that effective therapy contributed to mitigate the retinal atrophy of severe ITON. To our best knowledge, this is the first study using oxygen saturation of retinal blood vessels (SO_2_) measurement and optical coherence tomography angiography (OCT-A) to investigate the change of retinal vasculature in ITON individuals.

As an effective and safe therapy for ITON, ETOCD has been widely used as one of the most important treatments of ITON for decades [7–12]. However, the associated pathophysiological change and the underlying mechanism of ETOCD were not clear. It has been hypothesized that swelling of the optic nerve within the confined cavity of the optic canal compromises blood supply to the nerve within the canal, which exacerbates tissue ischemia and causes further damage to the injured optic nerve. The damage to the optic nerve due to intraneural edema alters microvasculature and interruption of direct axoplasmic transport. Also, vascular insufficiency resulted from the altered microvasculature might be an important pathogenic mechanism in other optic neuropathies [20, 23, 24], which implies that the abnormal hemodynamic state participates in the course of ITON, and the decompression surgery is effective by improving the retinal vasculature alteration. The spectrophotometric retinal oximetry and the optical coherence tomography angiography (OCT-A) are both noninvasive diagnostic tools to evaluate hemodynamic changes in eye diseases.

In this study, we assessed and analyzed the visual acuity (VA), visual evoked potential (VEP), oxygen saturation of retinal blood vessels, and the vessel density of optic nerve head and macular of 77 ITON patients who received ETOCD surgery and 18 ITON patients who underwent conservative therapies. Our results provided encouraging outcomes of ETOCD surgery, and suggested that it was an optimal choice for effective and safe therapy for ITON patients. Vision improvement is evidenced not only at clinical manifestation levels with multiple measurements of visual acuity but also at functional and pathophysiological and levels as evaluated by VEP, retinal oxygen saturation, and retina vessel density after ETOCD.

In the present study, 76.0% of patients have vision improved after ETOCD, and 33.3% of patients have vision improved after the conservative therapy, concordant with the literatures [25–27]. VEP was an important parameter of visual function, especially for the function of the optic nerve. In this study, it was found that VEP values were significantly improved after surgery in ITON patients. Among these main parameters of VEP, the latent period of N2 and P1 and the amplitude of P2 were sensitive to vision recovery in ITON patients.

Furthermore, the oxygen saturation of retinal arteries (SaO_2_) and veins (SvO_2_), as well as the differences between the arteries and veins (AVD), all increased in ITON patients after surgery. The oxygen saturation levels have been shown to associate with multiple ophthalmic conditions. For instance, SaO_2_ was significantly lower in retinitis pigmentosa patients [21]. In patients with an ischemic branch due to retinal vein occlusion, the occluded arteriole oxygen saturation increased compared to the saturation levels in vessels from the same quadrant in the contralateral eyes [28]. Zheng et al. also found decreased retinal arteriole saturation, a decreased difference in arteriovenous saturation, and a narrowing of retinal vessel diameter in highly myopic eyes [29]. In our study, we found that SaO_2_ and AVD, representing the use of oxygen by cells, were significantly higher in patients who treated with ETOCD, especially in the patients with VA improvement. Our findings were supportive that the increased oxygen saturation of retinal vessels and the better availability of oxygen in the retina may underlie the vasculature mechanism of VA improvement in ITON patients.

With the emergence of OCT-A, the inspection of retinal microvasculature updated our understanding to the pathogenesis and mechanism of several critical eye diseases, such as glaucoma, ischemic optic neuropathy, and congenital ophthalmopathy [30]. The OCT-A provides measurement of vessel density, which is evidential to the alteration of blood supply following traumatic impact to the optic nerve. To date, the research about the vascular changes in optic neuropathies with OCT-A has been scarce. Chan et al. [6] found that significant decrease in blood supply and oxygenation to the retina was associated with choroidal thinning in chronic ITON patients. Lee et al. [31] reported that in early ITON, a significant thinning of macular ganglion cell complex (GCC) was observed, which implied that GCC loss may participate in the development of ITON. In our study, we found the ETOCD surgery, especially the VA improvement after surgery, was associated with thicker retinas and better vessel density, which indicated that effective decompression surgery may mitigate the progress of retinal atrophy in severe ITON patients.

Our findings suggest that retinal hemodynamic changes, including the increased oxygen saturation of retinal vessels and the better availability of oxygen, were associated with vision recovery after effective treatment of ITON patients. Moreover, the effective treatment of ITON mitigates the progress of retinal atrophy of severe ITON.

## Data Availability

All data and materials support our published claims and comply with field standards.

## Code availability

All software application or custom code support our published claims and comply with field standards.
